# A shared approach to managing urinary tract infections in nursing homes improved perceived care quality, workload, and collaboration – a qualitative study

**DOI:** 10.1080/02813432.2025.2463455

**Published:** 2025-02-11

**Authors:** Sif Helene Arnold, Melissa M. W. Thomsen, Marius Brostrøm Kousgaard, Jette Nygaard Jensen

**Affiliations:** a The Research Unit for General Practice and Section of General Practice, Department of Public Health, University of Copenhagen, Denmark; b Department of Clinical Microbiology, Copenhagen University Hospital, Herlev and Gentofte, Copenhagen, Denmark

**Keywords:** Antibiotics, nursing homes, primary care, urinary tract infections, antibiotic stewardship, infection prevention, cross-sector collaboration

## Abstract

**Background:**

In 2019, around 4.95 million global deaths were linked to bacterial antimicrobial resistance (AMR). Managing urinary tract infections (UTIs) in nursing homes involves prevention, diagnosis, and treatment. This is often complex and cause excessive antibiotic use, increasing AMR. Infection prevention and antimicrobial stewardship (AMS) are complementary strategies for reducing AMR. Studies show that nursing home staff can safely reduce antibiotic prescriptions for UTIs using these strategies and that cross-sectoral collaboration with general practice is important in UTI management. However, the impact of combining infection prevention with AMS and general practice is unknown.

**Objective:**

To explore the perceived impact of a new cross-sectorial intervention combining prevention and AMS on UTI management in nursing homes.

**Methods:**

The intervention included a 3-h seminar for general practice and nursing home staff, and a reflection sheet to assess residents. We held 9 seminars in the Capital Region of Denmark in 2022 and conducted 15 semi-structured online and phone interviews with participants.

**Results:**

Our findings indicate that the intervention clarified workflows, encouraged nursing staff to adhere to agreements, and increased trust and respect between nursing homes and general practice. A reflection sheet was essential in linking planned changes to actual implementation. The sheet helped restructure UTI management, leading to perceived improved patient assessment and fewer UTI-related inquiries to general practice.

**Conclusion:**

Our findings suggest that the intervention had a positive impact on experienced care quality, workload, and cross-sector collaboration. However, physical attendance at the seminar limits the large-scale implementation of the intervention.

## Introduction

In 2019, 4,95 million global deaths were linked to bacterial antimicrobial resistance (AMR), and projections suggest that by 2050, annual deaths due to AMR will exceed those caused by cancer [1,2]. The prevalence of AMR is directly proportional to antibiotic use [3]. To safeguard clinical effectiveness, it is important to avoid unnecessary use of antibiotics.

In the elderly population, urinary tract infection (UTI) is the most common cause of antibiotic treatment [4]. However, this population is particularly vulnerable to the adverse effects of such treatment, including AMR, side effects and Clostridioides difficile infections [5,6]. Adding to this concern, many treatments for UTI are inappropriate, particularly among nursing home residents, compounding the risk of these adverse outcomes [7,8]. The problem of overtreatment of UTIs is especially prevalent in Danish nursing homes [9].

The World Health Organisation (WHO) recommends antimicrobial stewardship (AMS) and infection prevention as strategies to reduce inappropriate antibiotic treatment [10]. There have been several successful AMS interventions to reduce inappropriate antibiotic treatment for UTIs in nursing homes [11]. In Denmark, our research group designed and tested an AMS intervention that safely reduced antibiotic prescriptions for UTIs by improving knowledge and reflection skills among nursing home staff [12]. There is some evidence that infection prevention works to prevent AMR in nursing homes [13,14]. Consistent with this, the results from our regional quality improvement project suggest that infection prevention in combination with recognition of UTIs is very effective in decreasing UTIs in nursing homes [15]. Although the synergistic effects of integrating antibiotic stewardship and infection prevention have been highlighted, especially during the COVID-19 pandemic, they remain uncommon in interventions to combat AMR [16–18].

Infection management in nursing homes is often an interprofessional and cross-sectoral responsibility. Studies have found that cohesive collaboration between healthcare professionals and sectors supports effective UTI management and improves the quality of care [19–21]. In Danish nursing homes, it is a shared responsibility between healthcare professionals working in nursing homes and general practice [22]. The previously mentioned interventions performed by our research group were aimed solely at nursing homes [12,15] but an evaluation indicated that cross-sector alignment for UTI management practices in nursing homes is an important facilitator for implementation [23].

In light of this, we developed a new intervention that merged AMS and infection prevention aimed at both nursing homes and general practice. This study explores how the participants experienced the new intervention and its impact.

## Materials and methods

### The setting

We conducted nine intervention seminars in the Capital Region of Denmark in March, October, and November 2022. General practices and nursing homes from seven municipalities participated in the intervention. One municipality had so many participants that we had to organize two seminars. Additionally, we arranged a supplementary seminar as a ‘catch-up session’ for participants from various municipalities where there were not enough participants to justify a dedicated seminar.

Nursing home staff involved in UTI management includes temps, healthcare helpers (HCH), healthcare assistants (HCA), and nurses [24]. When nursing home staff members suspect an infection, they typically contact general practice. We have described the work functions of nursing home staff and workflow practices in UTI management in greater detail elsewhere [23]. Since 2017, the assignment of a specific general practitioner (GP) to each nursing home has been gradually introduced in Denmark [25]. In April 2023, the Danish Regions estimated that 83% of nursing homes had a designated GP [26].

### The intervention ‘Yes, no, maybe’

**Figure 1.**
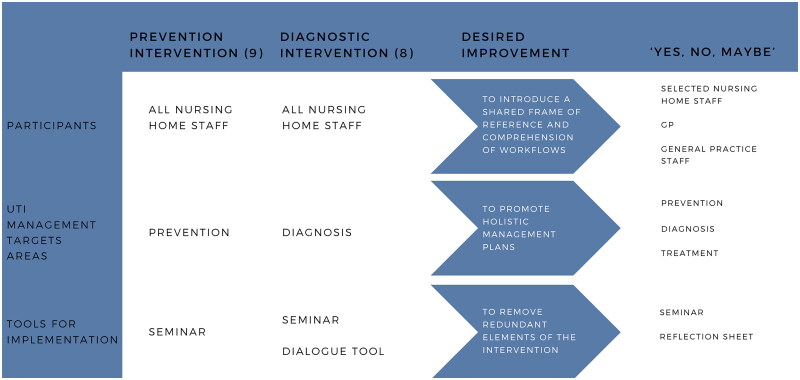
Similarities and differences of previous interventions (prevention intervention and diagnostic intervention) and ‘Yes, no, maybe’.

‘Yes, no, maybe’ combined two previous interventions focusing on prevention and diagnostics respectively, and brought general practice into active participation [12,15]. The new intervention actively engaged GPs, general practice staff, and selected nursing home staff. As part of this approach, UTI management tasks were expanded to three target areas: prevention, diagnosis, and treatment. The objective of reducing unnecessary antibiotic prescriptions for UTIs remained unchanged in the intervention. Figure 1 illustrates the similarities and differences between the two previous interventions and the new modified intervention.

The intervention had two main components: a reflection sheet and a 3-h seminar. The reflection sheet is a structured tool designed to guide health professionals through systematic assessment and reflection when a UTI is suspected in a nursing home resident. The reflection sheet is included in the supplementary material.

Each seminar started with an introduction to the agenda and objectives. Participants were divided into two groups: a) GPs and their staff and b) nursing home staff. Each group attended sessions tailored to their professional background, covering prevention, diagnosis, and treatment. The nursing home staff had a detailed infection prevention session, in which they discussed fluid intake, hand hygiene, intimate hygiene, Personal Protective Equipment (PPE), effective bladder drainage, hygienic KAD, and diaper management. The same infection prevention strategies, except for PPE, were briefly outlined for general practice. Both groups focused equally on diagnosing UTIs, supported by the reflection sheet. The general practice treatment session focused on antibiotic choice and urine culture interpretation, whereas this session for nursing home staff was short and focused on antibiotic resistance and urine sample collection. In the final part, both groups were reunited to discuss and use the reflection sheet in a hypothetical case of suspected UTI. For the discussion, the nursing home staff were paired with their designated general practice. Finally, they deliberated adjustments to their UTI management practices and agreed to follow-up plans.

### Study design

We used a qualitative research design based on semi-structured online and telephone interviews with healthcare providers from general practice and nursing homes who participated in the ‘Yes, no, maybe’ intervention.

### Study participants

We recruited 15 informants who had participated in ‘Yes, no, maybe’. These were six GPs, one general practice nurse, five nursing home nurses and three HCAs (Table 1). All informants were female and came from eight different municipalities. All but two informants had their collaborative counterparts present at the seminar.

**Table 1.** Characteristics of informants.

Informant #	Profession	Work facility	Municipality #	Collaborative counterpart present at seminar?
**1**	HCA	Nursing home	1	Yes
**2**	GP	General practice	2	Yes
**3**	Nurse	General practice	3	Yes
**4**	GP	General practice	4	Yes
**5**	GP	General practice	3	Yes
**6**	Nurse	Nursing home	1	Yes
**7**	Nurse	Nursing home	2	No
**8**	HCA	Nursing home	2	Yes
**9**	Nurse	Nursing home	5	Yes
**10**	HCA	Nursing home	4	Yes
**11**	Nurse	Nursing home	4	Yes
**12**	GP	General practice	6	No
**13**	Nurse	Nursing home	7	Yes
**14**	GP	General practice	8	Yes
**15**	GP	General practice	7	Yes

### Data collection and management

The interview guide covered several topics, including participants’ views on the seminar, their experiences with the reflection sheet, collaboration between general practice and nursing homes, and changes in daily practice since the seminar. The interviews were conducted approximately three months after the informants attended the seminar. Two research assistants conducted the interviews. They conducted the first three interviews together and then split the remaining interviews between them. The interviews were conducted using Microsoft Teams when possible, and over the phone in some cases to ensure flexibility and accessibility for the participants. Each interview lasted for approximately half an hour. They were recorded, transcribed verbatim, and anonymized.

### Analysis

We used Systematic Text Condensation (STC) for the analysis [27]. STC is a four-step iterative method. First, you identify themes, progress to code meaning units, then text condensation, and finally synthesize the condensations into analytical descriptions and concepts. After the initial reading of all the interviews, SHA, MT, and MBK agreed on seven preliminary themes. Two interviews were independently coded for meaning units by SHA, MT, and MBK. Code groups were subsequently established through discussion. SHA and MT collaboratively reviewed the remaining data and achieved consensus on the code groups before proceeding with the text condensation. Finally, the condensates were synthesized and organized into three category headings by SHA and reviewed by the remaining authors.

### Ethics

According to Danish legislation, interview studies are not subject to ethics committee approval. Participants at the seminars provided written consent, allowing us to reach out for an interview 3 months after the seminar. GPs were reimbursed for participation.

## Results

The results of our analysis of the experienced impact of the intervention on UTI management is presented in three category headings. The first category heading relates to the new collaborative space created at the seminar. The second category heading concerns how the reflection sheet supported the implementation. The final category heading addresses how the intervention was perceived to improve care and cross-sectoral collaboration.

All participants considered the seminar a suitable way to introduce the principles, concepts, and tools in ‘Yes, no, maybe?’ All informants expressed that attending was valuable for UTI management. For the nursing home staff, the seminar provided new insights regarding what constitutes a UTI, while for participants from general practice, the informational part of the seminar primarily served to reinforce existing clinical knowledge. Overall, the intervention seemed to be very well received.

### The seminar created a new collaborative space

According to the informants, discussions at the seminar often resulted in agreement about changes in work routines. To improve prevention, nursing staff should increase their focus on monitoring hydration levels, implement infection prevention practices, and perform bladder scans for residual urine. During the seminar, agreements to improve diagnosis included nursing home staff committing to routinely use the reflection sheet, to always consult a nurse or assistant before contacting general practice and dispatching urine samples, and to consistently use boric acid glasses for urine samples. General practice participants agreed to support these changes by reminding nursing home staff of prevention measures, responding to diagnostic inquiries in accordance with the reflection sheet, and supplying boric acid containers. Thus, the most significant changes were meant to take place at the nursing home before the urine sample was sent to general practice.

The informants emphasized that it was advantageous to have their collaborating counterparts from the nursing home and general practice present at the seminar because it promoted agreements about the practice changes needed to implement new insights gained from the seminar. A nurse conveyed a common sentiment about the importance of these direct, in-person interactions:

We got to sit down and get some answers … it definitely made it more clear who is responsible for what and when. (Informant #13, Nursing home nurse)

By contrast, one nursing home struggled after the seminar because their designated general practice had not participated. Although the nursing home nurse briefed the practice on the seminar and presented the reflection sheet, this was not sufficient to align the UTI management practices.

I wish that our doctors had been involved, because I can feel that it takes away some of the motivation to do this work if we end up being told that we should collect a urine sample for C&S [Culture and Sensibility testing] when we contact the GP [for advice] although we don’t suspect UTI. (Informant #7, Nursing home nurse)

Thus, it seems that the seminar created a missing space for clarifying workflow practices and mutual expectations, even though collaboration already existed. This also suggests that having general practice physically present at the seminar, especially the GP, facilitated the alignment of UTI management practices by unifying understanding and soliciting support from general practice for nursing homes’ efforts.

### The reflection sheet facilitated the transition from planned workflow changes to implementation

All participants agreed that the reflection sheet had improved UTI management. At the individual level, participants reported that the reflection sheet functioned as a memory aid and recipe for a systematic assessment of the patient, in accordance with its purpose of enhancing the quality and consistency of UTI management. Thus, it helped the individual user know which observations to look for, which reflections to engage in, and what information to pass on. The way the reflection sheet distilled a thought process into a specific procedure—a “cookbook”—was considered especially valuable for nursing home staff, who did not have the professional training to reflect on the clinical diagnosis in a systematic way. The sheet did indeed seem to foster increased reflection on the observed symptoms for this group.

… it also somewhat forces you to think about whether you have observed any pain, whether you have observed that it stings when they urinate, or something like that. It forces us to consider whether there are any, whether they [the nursing home residents, red.] express any symptoms. (Informant #1, Nursing home HCA)

The reflection sheet, originally developed for nursing home staff, also proved useful for some experienced GPs in reevaluating their understanding of UTIs in the elderly:

…I think you become quite knowledgeable [when you use the reflection sheet, red.]. Even after many years, one might think it’s probably a UTI causing him or her to behave differently. But then, when you go through this flowchart, I am still surprised to see it says ‘NO, there is no urinary tract infection’ [laughs], so yes, I still use it. (Informant #5, GP)

This GP found it helpful in recognizing when to consider an alternative diagnosis to UTI. Thus, the reflection sheet functioned as a debiasing tool, also for GPs, to critically appraise clinical thinking among the participating healthcare providers.

In the interprofessional and cross-sectoral collaborations there were more uses of the reflection. First, during the seminar, nursing staff and GPs agreed that nursing staff with the highest clinical education should be consulted before reaching out to general practice or dispatching a urine sample. This decision established a structured hierarchy to ensure quality and consistency in communication. The individual serving this function was responsible for explaining their clinical reasoning to colleagues, with the reflection sheet providing essential support for these professional dialogues:

So, the reflection sheet made sense in terms of providing structure. Now it’s easier for me to explain why we do what we do. Therefore, it’s easier for me to convey my knowledge to my colleagues. (Informant #9, Nursing home nurse)

Together, the agreement and the reflection sheet defined a ‘gatekeeper role’, in which nursing home nurses or HCAs authorized contact with general practice while simultaneously reiterating the clinical reasoning in UTI management.

Second, an additional ‘gatekeeper role’ emerged in general practice. When the nursing staff reached out, GPs and their staff used the reflection sheet to assess the relevance and quality of the inquiry. If information was missing or the reflection sheet was not used, the urine samples or other requests were rejected. This ‘gatekeeper role’ was usually handled by general practice staff over the phone, protected the GP’s time. In addition, GPs also used the reflection sheet this way themselves during nursing home visits.

Third, the reflection sheet heightened awareness of the different kinds of information that went into the diagnostic decision and who provided it. A key message at the seminar was that the GPs assessments of a resident’s condition depend on the quality of the clinical information gathered by healthcare professionals close to the patient. The reflection sheet ensured that more relevant and complete information reached the GP.

… well then, it’s not certain that the correct information eventually reaches our table, and it’s this information we end up making a decision on. But I think this reflection sheet makes it easier and better. (Informant #4, GP)

The use of the reflection sheet also heightened awareness of where the different types of information came from. Being close to the patient, nursing staff could add insights into daily changes in residents’ lives, which might impact their wellbeing. Having access to the patient’s medical history, the GP could contribute to broader diagnostic considerations. This enriched the diagnostic process, resulting in a more nuanced understanding of residents’ health issues and reducing the risk of misdiagnosis:

So it turned out that actually quite a few of them, we found out, had something completely different going on [than UTI], or not many of them, but some. (Informant #4, GP)

The reflection sheet facilitated the integration of previously disparate information from nursing homes and general practice, leading to a more comprehensive assessment of residents.

The reflection sheet served many functions for individual users and in interprofessional and cross-sectoral collaboration. Some of the uses were suggested at the seminar or were inherent to the reflection sheet. All the individual and interprofessional uses were intended, whereas the cross-sectoral uses emerged through individual agreements between nursing homes and general practice and their use of the reflection sheet. Overall, the reflection sheet helped transition planned workflow changes from the seminar into practice.

### Restructuring UTI management practices improved the perceived quality of care and cross-sectoral collaboration

Three months after the seminar, many informants from general practice and nursing homes reported a positive impact on their work and collaborative relationships. Some spoke of a ‘before and after’.

Informants mentioned that after the seminar, previously ignored agreements were maintained. In one nursing home, the GP initiated efforts to reduce dipstick use. Previously, when dipsticks were banned, they were autonomously reinstated by staff. However, 3 months after the seminar, with dipsticks made less accessible, they remained unused. Other successfully implemented agreements included consulting a nurse about signs and symptoms and seeking the GPs opinion before dispatching a urinary sample.

Nearly all informants perceived improvement in the quality of UTI management after the seminar. Improvements were linked to increased conscious deliberation at each step of prevention, diagnosis, and treatment. According to the informants, infection prevention and rehydration were increasingly used at nursing homes, and the nursing staff’s tendency to send a urine sample for testing at minor signs of a change in patients was tempered, giving way to a more reflective approach to managing changes in the patient’s condition.

… our workflow at the nursing home was just like, well, we just do it [take the urine sample], and then we rush over with it [to the GP clinic], where instead you could say – now there’s a lot more reasoning behind it. (Informant #11, Nursing home nurse)

With more careful prevention and diagnosis, GPs experienced that suspicions of UTI were now more frequently managed efficiently without general practice involvement.

All the unnecessary stuff – which the sheet should help with – like behavioral changes and all those grey area things where there aren’t actually urinary tract symptoms – we clearly experience that they sort that out there [at the nursing home], and they do it really well. (Informant #15, GP)

The GPs experienced that the improved understanding among nursing staff about evaluating suspected UTIs reduced the pressure on GPs to prescribe antibiotics or to explain clinical decisions. GPs also tended to wait for urine culture results and re-check symptoms before starting treatment. The general perception was that managing UTIs had become less time-consuming for the GPs.

Since most of the changes resulting from the seminar took place at the nursing homes, this might have increased the workload of the nursing home staff. However, the informants did not experience an increased workload.

Well, I actually think in some way it occupies us a bit less – it’s a bit funny, isn’t it. but because we haven’t really had, we’ve been able to quickly dismiss the suspicions that were there, or found something else, before we just called the GP or took a dipstick, so I think it’s as if there’s less focus on it, in a positive way. (Informant #9, Nursing home nurse)

Hence, it seemed that the more systematic approach allowed quicker dismissal of suspected UTI, leading to fewer contacts with general practice. When GPs were consulted, they found that the clinical information was more relevant and that the cases more often were “true” UTIs. The perceived increased professionalism in the inquiries led to greater respect for nursing staff in their interactions with the GPs.

They earn deeper respect from me if they have acted in a correct professional way. This is true in all areas, where you see people taking things seriously, treating them with importance, using them, and following the rules we collectively established at the seminar. (Informant #5, GP)

This sense of increased alignment and trust was also evident to the nursing home staff, who felt that their clinical assessments were now more recognized and valued.

Overall, the participants perceived that the quality of care in UTI management increased because prevention, diagnosis, and treatment became more systematic and deliberate. As a result the participants, and especially the GPs, experienced a reduced workload concerning UTI. Furthermore, the collaborating participants had developed an increased sense of trust and respect of each other across sectors.

## Discussion

This study explored the perceived impact of an intervention that merged infection prevention with AMS and included general practice to improve UTI management in nursing homes. Our findings indicate that the changes to the intervention created a new space for clarifying workflows and improved perceived quality of care and collaboration between nursing homes and general practice. The reflection sheet was essential in linking the planned changes to the actual implementation. It helped restructure UTI management, leading to perceived improved patient assessment and fewer UTI-related inquiries to general practice.

The ‘Yes, no, maybe’ intervention grouped UTI management tasks into three areas, outlining clear roles for nursing home staff and general practice. The nursing home staff focused on prevention, general practice on initiating treatment, and both shared diagnostic responsibilities. This approach integrated infection control with AMS, promoting mutual understanding of each healthcare professional’s value in UTI management. The reflection sheet streamlined this process and established a common language. Consequently, nursing staff often managed suspected UTIs more independently, while general practice played a vital, albeit indirect, role by acknowledging staff contributions and using the sheet to maintain systematic management.

The designated GP arrangement has been praised for improving collaboration between general practice and nursing home staff [28]. Therefore, it was surprising that participants unanimously emphasized the shared discussion at the end of the seminar to create a new space for clarifying workflow practices and mutual expectations as important. On the other hand, cross-sector collaborations in healthcare are often problem-focused and occur in time-scarce environments [29]. Therefore, the intervention may have provided an opportunity to collectively reflect on the general principles for improving UTI management that the designated GP arrangement did not provide.

Nurses’ roles in antimicrobial stewardship, though only recently recognized, are diverse [30]. Currie et al. found that nurses often underestimate their own impact or hesitate to question doctors’ decisions regarding antibiotic stewardship [31]. Müller et al. emphasized the need for interventions to clarify nursing home staff’s roles and foster relationships with general practice so that care and treatment issues can be safely raised [32]. This study indicates that the ‘Yes, no, maybe’ approach met these objectives.

The seminar facilitated dialogue between the nursing home staff and general practice, clarifying roles, responsibilities, and workflows. Such meetings have enhanced teamwork in other nursing homes [32]. Our analysis suggests that GPs’ involvement was key to strengthening commitment from both groups. While the exact mechanisms are unclear, the professional hierarchy among health professionals in primary care, often positioning the GP at the top, may also play a role [33,34].

We found that the intervention, particularly the reflection sheet, led to the emergence of two ‘gatekeeper’ roles. The first ‘gatekeeper role’ appeared at the nursing home, when the nurse or the HCA authorized other staff to contact general practice. These results corroborate our previous findings regarding the implementation of the reflection sheet [23]. The second ‘gatekeeper role’ arose within general practice and set the bar for inquiries to be sufficiently explored by the nursing home before the GP should act on them. A similar ‘gatekeeper role’ was observed when the designated Danish GP program was evaluated [28]. Thus, the reflection sheet seemed to fit into this known organizational pathway within the cross-sector collaboration and add another pathway within the nursing home. Collectively, these two ‘gatekeeper roles’ reduced the perceived number of inquiries to the GPs.

## Strengths and limitations

In this study, we used qualitative interviews to explore the experiences of participants with a new modified intervention for improved UTI management. Strengths of the study included a) participant triangulation as we recruited both GPs, nurses, and HCAs from general practices and nursing homes, b) researcher triangulation where several researchers with different professional backgrounds contributed to the various phases of the study, e.g. coding and interpretation, and c) the fact that three of the authors (SHA, MBK and JNJ) had previous research experiences in the field of UTI management interventions. However, the study also has some limitations.

First, while the sample included the key actors in UTI management, it lacked representation of HCHs and temps, who are also involved in some of the work targeted by the intervention, and hence, we do not know how these groups perceive the intervention and its implications. Further, all our informants turned out to be female (despite our efforts to include diverse demographics), potentially sidelining experiences or perspectives specific to male healthcare providers (although they constitute a small minority in nursing homes).

Second, concerning the use of interviews, we conducted the interviews using Microsoft Teams and telephone calls to reduce barriers to participation. However, the virtual and brief format may have restricted our understanding of nonverbal cues and other intricate nuances that are typically apparent in longer in-person interactions. Also, we conducted interviews three months after the seminars, granting participants time to gain experience with implementation of the intervention, but this gap may have introduced recall problems, where fading memories may lead to omissions or distortions in recollections. Further, while qualitative interviews are appropriate for generating knowledge about the experiences of the intervention participants, ethnographic observations (which we did not employ due to resource constrains) could have provided a more realistic and detailed impression of how the intervention affected diagnostic and collaborative practices, and thus have increased the internal validity of the study.

Finally, SHA and JNJ designed and delivered ‘Yes, no, maybe’, and JNJ, SHA, and MBK developed parts of the previous interventions. Although we believe that a deep understanding of the intervention enhances our analysis and interpretation, we recognize that there is a risk of unintentionally focusing on the positive aspects of the intervention’s impact, while overlooking potential shortcomings.

In terms of the transferability, or external validity, of the findings, the study involved eight municipalities from the Capital Region of Denmark. Municipalities in the Capital Region are slightly more urbanized than other regions. While the primary care sector here may have easier access to healthcare professionals, three of the municipalities were predominantly rural, representing considerable variations between urbanization, population density and even healthcare access. Despite these variations, the informants from nearly all municipalities repeatedly confirmed that participants had developed a shared framework for managing UTIs. Therefore, we believe the findings are relatively transferable to nursing homes in other parts of Denmark and to primary care settings in other countries with similar healthcare systems, particularly those where general practice plays a central role in supporting nursing homes.

## Conclusion

Our findings suggest that the ‘Yes, no, maybe’ intervention had a positive impact on health professionals work routines and workload, as well as on the collaborative relations between nursing homes and general practice. With growing healthcare demands and the increased need for cross-sector collaboration, ‘Yes, no, maybe’ outlines a promising direction within the area of UTI management. Indeed, using a shared frame of reference that combines infection prevention with AMS may be a transferable strategy for the cross-sector management of other diseases such as respiratory tract infections. However, the seminar’s need for physical attendance limits large-scale implementation of the intervention. Thus, a different method of intervention delivery may be needed for the widespread diffusion and implementation of the ‘Yes, no, maybe’ model of UTI management.

## Supplementary Material

Supplemental Material
