# Supplementary figures and images for: A shared approach to managing urinary tract infections in nursing homes improved perceived care quality, workload, and collaboration – a qualitative study
Source: Scand J Prim Health Care. 2025 Feb 11;43(2):500–9. doi: 10.1080/02813432.2025.2463455 (PMC12090265; doi:10.1080/02813432.2025.2463455)

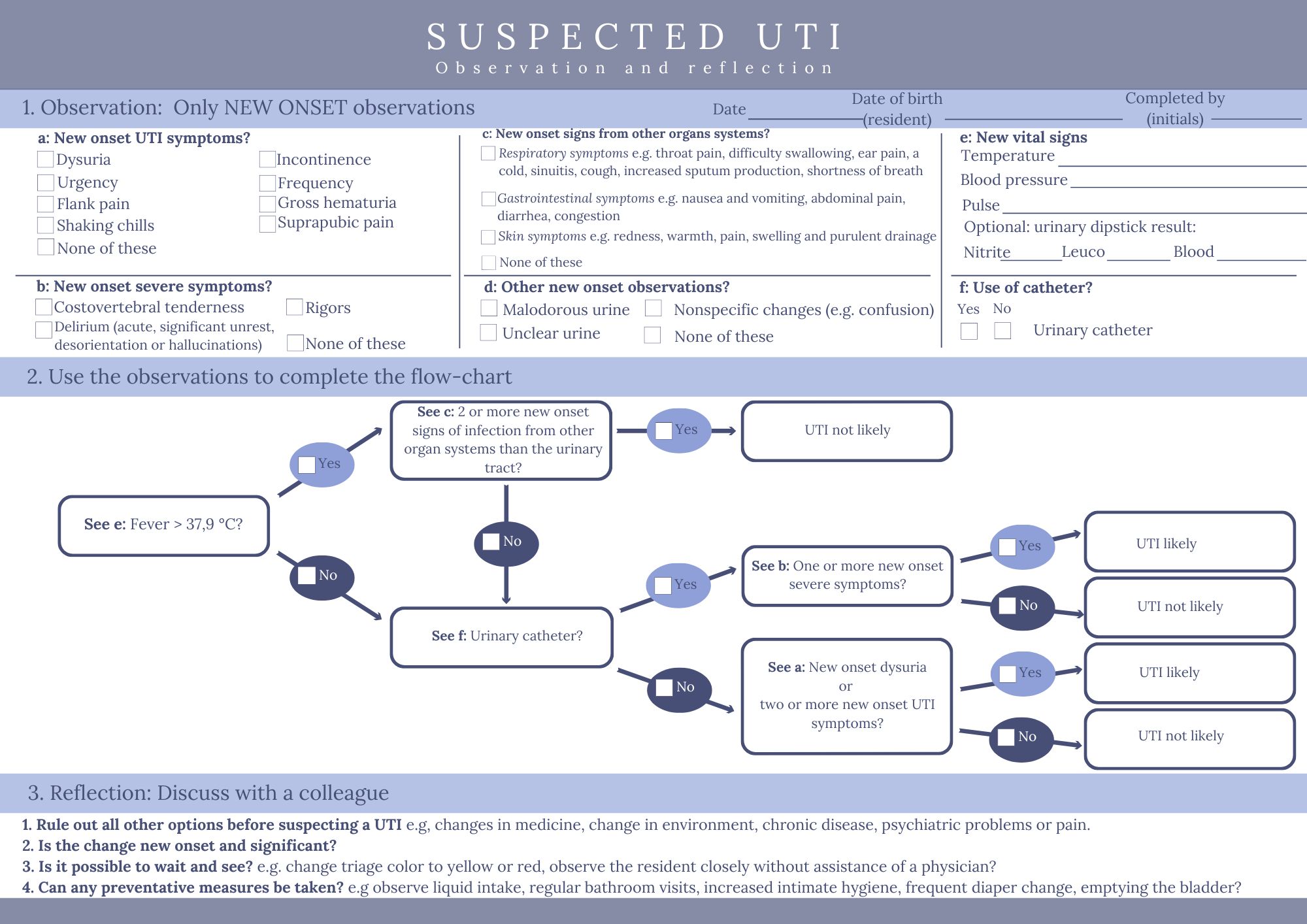

Supplement: Supplemental Material [file IPRI_A_2463455_SM3802.jpg]
